# Chemoembolization of liver cancer with drug-loading microsphere 50-100μm

**DOI:** 10.18632/oncotarget.14281

**Published:** 2016-12-27

**Authors:** Jun-Hui Sun, Guan-Hui Zhou, Yue-Lin Zhang, Chun-Hui Nie, Tan-Yang Zhou, Jing Ai, Tong-Yin Zhu, Wei-Lin Wang, Shu-Sen Zheng

**Affiliations:** ^1^ Division of Hepatobiliary and Pancreatic Interventional Center, First Affiliated Hospital, College of Medicine, Zhejiang University, Hangzhou 310003, P.R. China; ^2^ Department of Ophthalmology, Second Affiliated Hospital, School of Medicine, Zhejiang University, Hangzhou 310009, P.R. China

**Keywords:** chemoembolization, TACE, liver cancer, hepasphere microsphere, drug eluting microsphere

## Abstract

Transcatheter arterial chemoembolization (TACE) is the mainstay of treatment for unresectable hepatocellular carcinoma (HCC). The efficacy of conventional TACE (cTACE) in liver metastases is not satisfactory, which might be due to the fact of embolic material used. Recently, as a new type of drug-loading microsphere, HepaSphere has been introduced in China. In this study, there were total 30 patients (18 males and 12 females) with liver cancer underwent embolization with Hepasphere microsphere. And a total of 44 TACE procedures were performed using 50-100μm HepaSphere. There were 16 patients diagnosed as HCC and 14 patients as liver metastases. The follow up period ranged from 3 to 15 months (median 10 months). Response rates were calculated on intention-to-treat basis. One month after TACE, total objective response was 63.3% and disease control rate was 86.7%. No severe complication (such as infection, liver abscess, abdominal bleeding, tumor rupture) was observed. In conclusion, chemoembolization with Hepasphere microsphere may be a safe and possibly effective palliative treatment for patients with liver cancer.

## INTRODUCTION

Transarterial chemoembolization (TACE) is a well-recommended treatment for unresectable intermediate stage hepatocellular carcinoma (HCC) patients [1–3]. Unfortunately, the efficacy of conventional TACE (cTACE) in liver metastases is not satisfactory, and this might be due to the embolic material used. Lipiodol is the most common material used, and plays an important role in any cTACE procedure. It can be mixed with chemotherapeutic drugs, acting like a carrier, to enable the concentration and retention of the chemotherapeutic agent into the tumor.

HepaSphere microspheres (Merit Medical Systems, Inc., South Jordan, UT, USA) are soft, with a high conformability, and can be loaded with doxorubicin. They have been used for number of years, but have just recently been introduced in China. These small, nonabsorbable, doxorubicin-loaded microspheres can be more distally embolized, releasing the chemotherapeutic agent in a controlled and prolonged manner into the tumor, with lower systemic toxicity. Initial clinical results have been published, and show a good tumor response and safety profile [4–6]. Thus far, there have been no studies in the clinical literature about the use of HepaSphere microspheres in liver cancer in China. The aim of this study was to present the clinical efficacy and safety of HepaSphere microspheres in liver cancer management in China.

## RESULTS

The technical success was 100%, with a total of 44 HepaSphere procedures being performed in the 30 patients. There were 16 patients with HCCs and 14 patients with liver metastases, with the following primary tumor sites: colorectal, bile duct, neuroendocrine, gallbladder, pancreatic, lung, and gastric. The overall median follow-up period was six months, ranging from three to 15 months.

**Figure 1:**
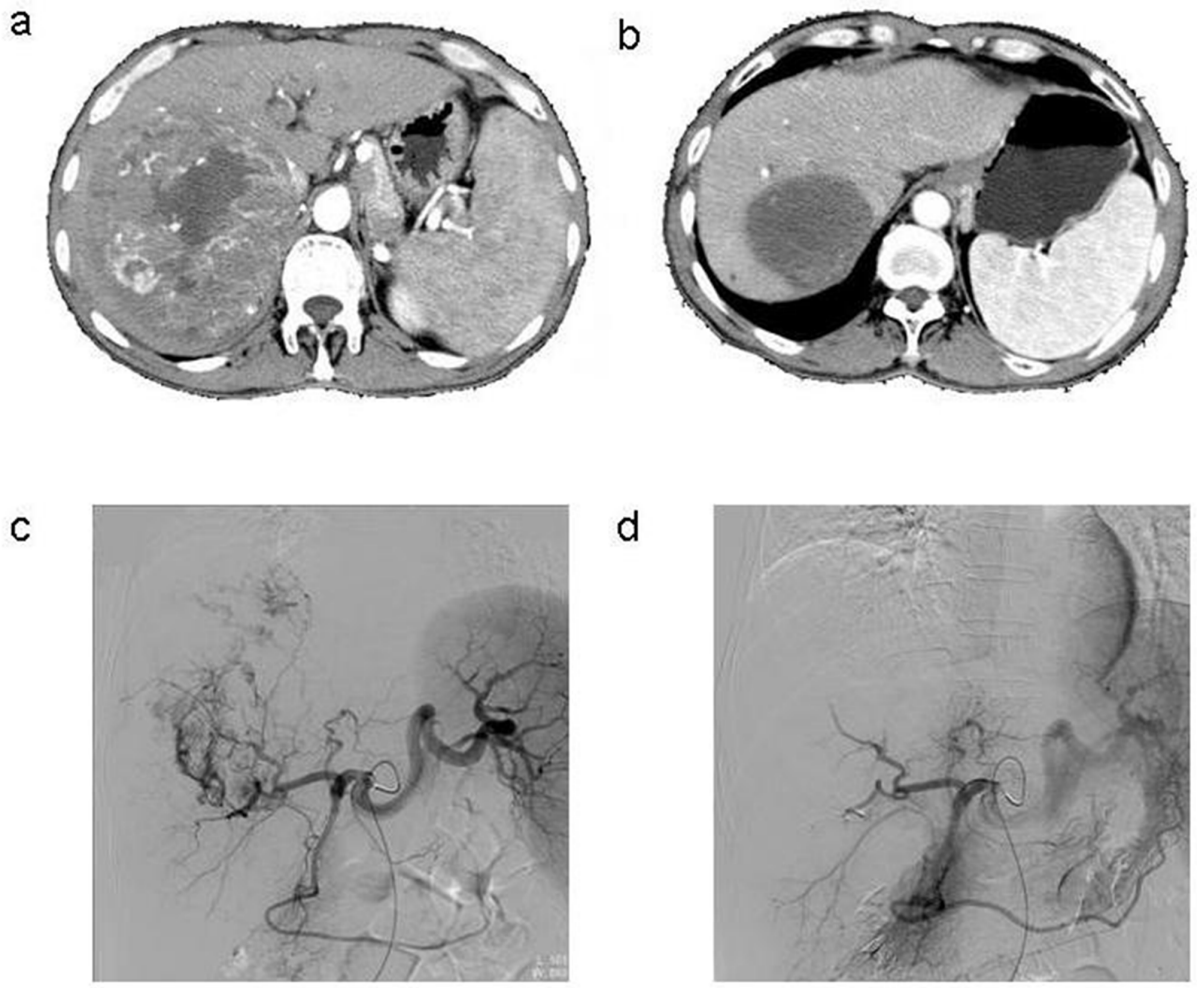
**a**. Contrast-enhanced CT scan done before the procedure in the patient with HCC, **b**. Enhanced CT showed complete tumor necrosis 6 months after three TACE sessions, **c**. Common artery angiography of the patient with HCC during 1^st^ TACE, selective catheterisation of the pathologic branch of right hepatic artery supplying the tumor, **d**. After chemoembolization angiography with 1^st^ TACE.

**Figure 2:**
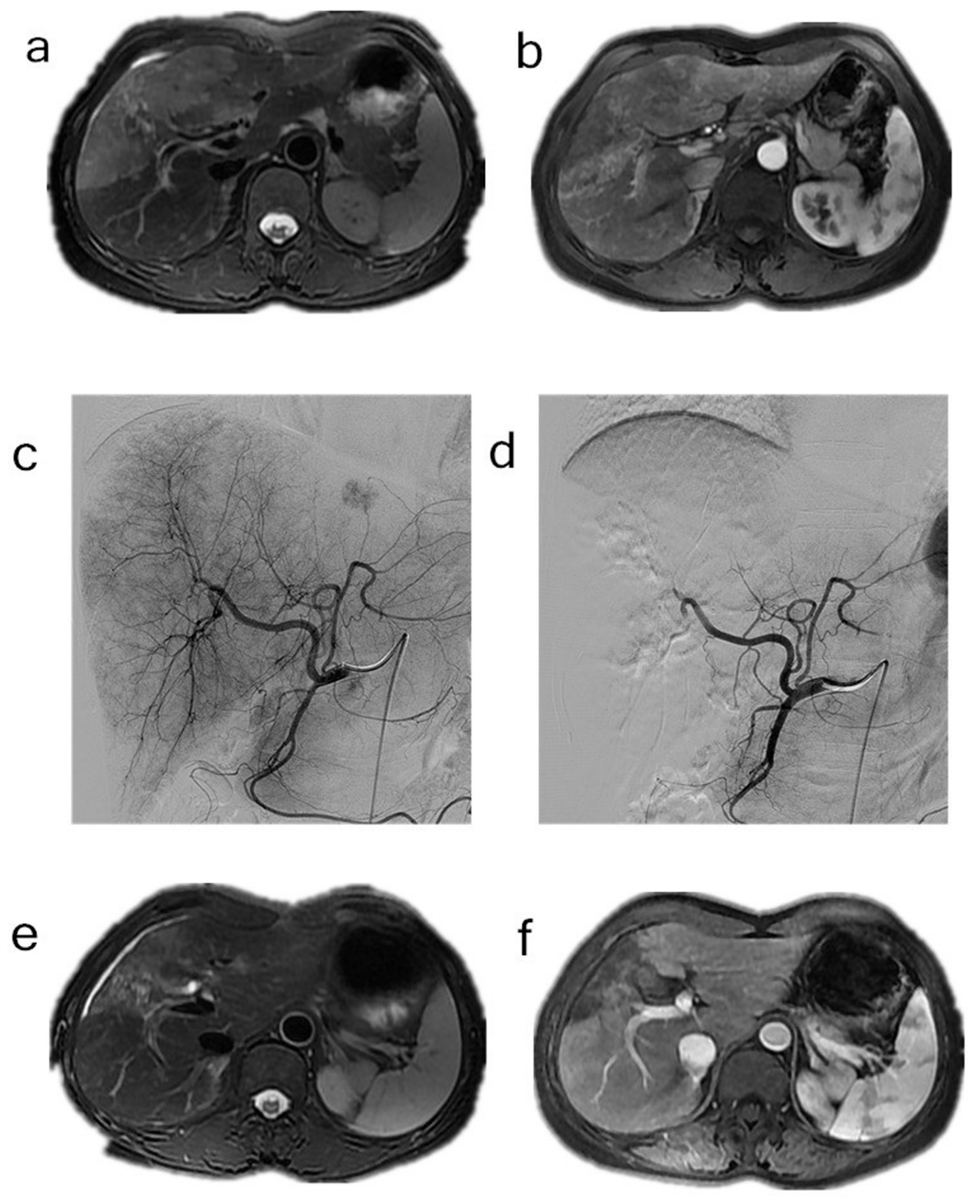
**a-b**. Contrast-enhanced MRI and CT scan done before the procedure in the patient with cholangiocarcinoma, **c**. Common hepatic artery angiography of the patient with cholangiocarcinoma, **d**. After chemoembolization angiography with extraction of pathologic vascularization, **e-f**. Gadolinium contrast MRI control 6 months after TACE, extraction of the vascularization of the tumor.

The tumor response was evaluated using CT/MRI according to the mRECIST criteria [7]. Following the HepaSphere microsphere treatment, two patients (6.7%) had complete responses (CR) (Figure 1), 17 (56.7%) had partial responses (PR) (Figure 2), seven (23.3%) had stable disease (SD), and four (13.3%) had progressive disease (PD). The total objective response was 63.3%, and the disease control rate was 86.7%. The pre-TACE and post-TACE tumor indicator details are given in Table 1, and many of the tumor markers had decreased one month postembolization.

**Table 1:** The details of tumor indicators pre-TACE and post-TACE

Patient number	Type of tumor	Type of tumor marker	Pre-TACE	1m Post-TACE
1	HCC	AFP	6373.3	2332.1
2	HCC	AFP	204.1	3.5
3	metastatic gallbladder tumor	CA199	12000	12000
4	mCRC	CEA	15.4	4.3
5	mCRC	CEA	133.1	72.6
6	HCC	AFP	4031.9	15.2
7	HCC	AFP	2532.3	471.2
8	metastatic neuroendocrine tumor	CA199	67.1	50.9
9	metastatic cholangiocarcinoma	CA199	12000	230.1
10	metastatic lung tumor	CA199	13.4	12.5
11	mCRC	CEA	50.5	33.1
12	metastatic gastric cancer	CA199	7.2	4.1
13	HCC	AFP	62.8	30.5
14	HCC	AFP	193.5	39.9
15	HCC	AFP	1.3	0.9
16	HCC	AFP	339.9	133
17	HCC	AFP	7.8	4.9
18	HCC	AFP	6.7	5.6
19	HCC	AFP	908.3	81.3
20	metastatic cholangiocarcinoma	CA199	40.9	23.1
21	metastatic pancreatic carcinoma	CA199	1113.8	58.1
22	HCC	AFP	2.7	5.8
23	HCC	AFP	3	2.6
24	HCC	AFP	26	15.3
25	metastatic gallbladder tumor	CA199	6.3	11.9
26	metastatic neuroendocrine tumor	CA199	3.3	2.9
27	metastatic neuroendocrine tumor	CA199	3	3.4
28	metastatic pancreatic carcinoma	CA199	153.6	8.6
29	HCC	AFP	91.9	59.3
30	HCC	AFP	2.3	1.6

Grade 2 toxicities (NCI-CTC) occurred in 43.3% (n=13), and grade 3 toxicities occurred in 26.7% (n=8) of all of the courses in terms of the liver function post-TACE (Table 2). No reversible leukopenia or thrombocytopenia occurred in any of the patients; however, mild fevers (grade 1, n=11) and abdominal pain (grade 3, n=7) were observed in some of the cases. Three of the patients had moderate abdominal pain and were treated with pethidine hydrochloride. Overall, none of the patients had any severe complications, such as bile duct infections, liver abscesses, abdominal bleeding, tumor ruptures, gastrointestinal bleeding, marrow suppression, or myocardiotoxicity.

**Table 2:** Change of liver function for patients with pre-TACE and post-TACE

Liver function	Pre-TACE	3d Post-TACE (P)*	1m Post-TACE (P)#
ALB (g/L)	41.7±3.54	36.9±5.82 (0.12)	39.9±4.68 (0.22)
ALT (U/L)	30.8±11.6	88.1±52.9 (0.001)	33.8±10.2 (0.17)
AST (U/L)	37.3±15.3	122.1±72.5 (0.002)	41.2±15.3 (0.19)
TB (μmol/L)	12.8±4.52	18.3±7.65 (0.001)	13.2±6.22 (0.17)

* 3d Post-TACE vs Pre-TACE, #1m Post-TACE vs Pre-TACE

## DISCUSSION

HCC is the fifth most common form of cancer, and the third leading cause of cancer-related death worldwide. Approximately 50% of the world HCC incidence is found in China, where it is the second leading cause of cancer-related death [8]. While resection is the first-line curative treatment for liver cancer, the majority of patients are not candidates for resection; therefore, TACE is the mainstay of treatment for unresectable intermediate stage HCC patients. TACE has been clinically proven to prolong overall survival, while showing potential benefits for a patient's quality of life.

Embolic material plays an important role in a TACE procedure. The cTACE technique requires the transarterial infusion of lipiodol and a chemotherapeutic agent, such as doxorubicin or cisplatin, into the hepatic artery, which can be followed by the embolic material. However, there are two major drawbacks to this technique: (1) incomplete lipiodol deposition in the tumor, leading to a poorer tumor response [9], and (2) most chemotherapeutic agents are hydrophilic, while lipiodol is not. This mixture would be transient, allowing for a quick release of the chemotherapeutic drug into the systemic circulation, leading to an increase in adverse events and a decrease in the local regional response. In liver cancer management, the slow release of a chemotherapeutic drug and prolonged exposure in the tumor, while lowering the systemic response, would result in a better response. Therefore, a new microsphere was developed to accommodate the need for a better embolic material with drug-eluting ability. There are two kinds of drug-eluting microspheres: HepaSphere and DC Bead [6, 10]. These microspheres could carry doxorubicin to the tumor and increase the intratumoral concentration.

When compared with lipiodol, which is commonly used in cTACE, HepaSphere microspheres have a superior safety profile and could carry a larger amount of the chemotherapeutic drug that can be released in a slow and controlled manner. The doxorubicin is released by the microspheres for a period of one month after the embolization [11]. This could provide more consistent results and facilitate the standardization of the TACE procedure.

Since the drug-eluting TACE procedure does not require the use of lipiodol, the evaluation of the tumor is not hindered by lipiodol retention, and it can be performed properly using Modified Response Evaluation Criteria In Solid Tumors (mRECIST) criteria [7]. With regard to the size of the embolic material, there is no recommendation, and it should be based on the vascularity, vessel size, and tumor anatomy. Some studies using drug-eluting embolic materials have shown that smaller calibers of microspheres are attractive, because they can achieve more distal embolization [12–15]. Only diameters <300 μm penetrate deep into the tumor microvasculature, according to the study by Lee et al. [16]. Distal embolization is desirable to avoid hypoxia-induced neoangiogenesis [17–19]. In our study, the HepaSphere microspheres were 50-100 μm in the dry state, and this was the smallest size available, since the 30-60 μm HepaSphere microspheres have not yet been introduced in China. The size the 50-100 μm HepaSphere microsphere in vivo is 200-400 μm after loading with doxorubicin; therefore, it is slightly larger than the DC Bead microsphere, which is 100-300 μm [11].

Several previous studies have shown good tumor responses in HepaSphere HCC treatment. In one study conducted by Malagari et al. in 2014 [6], the objective response was 68.9% and the one year survival was 100%, with a median follow-up period of 15.6 months. For advanced unresectable HCC, Kirchhoff et al. used degradable starch microspheres and iodized oil for embolization, and the response rates were: PD 9%, SD 55%, PR 36%, and CR 0% [20]. The overall one, two, and three-year survival rates were 75%, 59%, and 41%, respectively, and the median survival was 26 months.

Sorafenib is recommended for advanced stage HCC treatment. In a Phase II trial of sorafenib conducted by Pawlik, combined with concurrent transarterial chemoembolization and drug-eluting beads for HCC treatment, the results showed a good disease control rate of 95%. This suggests that combining cTACE and sorafenib could lead to better disease control with an acceptable complication rate [21]. Most liver metastases are hypovascular in nature, and the cTACE treatment response remains unsatisfactory, with possible complication development, like infection. In the study by Jarzabek et al., doxorubicin loaded HepaSphere microspheres were used as the liver metastasis treatment. The objective response rate was 26.7%, and the disease control rate was 60.1%, indicating that HepaSphere microspheres are a potential treatment for liver metastases [5]. These patients showed liver metastases from colorectal, cholangiocarcinoma, gastrinoma, gallbladder, pancreatic, gastrointestinal stromal, lung, kidney, breast, and larynx primary tumors. Another study conducted by Huppert et al. using irinotecan loaded HepaSphere microspheres in metastatic colorectal cancer (mCRC) showed the HepaSphere microspheres to be safe and effective in mCRC treatment [22]. The preliminary results from our study also showed a promising result with the use of HepaSphere microspheres in liver cancer treatment. The total objective response rate was 63.3%, and the disease control rate was 86.7%, which seemed to be superior or comparable with the results of the studies in which drug-eluting devices of 100-300 μm were used [13, 23].

Overall, drug-eluting TACE using HepaSphere microspheres is safe and well tolerated. The liver enzymes were slightly increased after embolization in all of the patients, but returned to normal 3-4 days later, a pattern that was also observed in other drug-eluting embolic studies [13, 15, 24–26]. This happened frequently in the cTACE procedures. More than 90% of those patients that underwent cTACE treatment exhibited postembolization syndrome (PES). However, only some of the patients treated with HepaSphere microspheres showed mild PES, and the severity was much lesser than those undergoing cTACE. Based on previous studies of drug-eluting TACE with DC Bead microspheres, complications occurred in 4.2%-11.4% of the cases, including gastric ulcers, liver failure, and cholecystitis. The treatment-related mortality was 0%-3.7%, mainly due to tumor rupture, liver failure, liver abscess, and cholecystitis [24, 25]. None of our patients treated with HepaSphere microspheres showed these complications.

The limitations of our study were that it was a single center study with a small patient number and a short follow-up period, with diverse patient characteristics and tumor backgrounds. Further investigations are required to evaluate the long term efficacy in a larger cohort or a randomized control trial. Despite these limitations, the use of HepaSphere microspheres in liver cancer management may be a safe, well-tolerated, and effective treatment modality, which has been shown to have a good objective response and disease control rate in both hypervascular and hypovascular tumors.

## MATERIALS AND METHODS

### Patients

Thirty patients from the First Affiliated Hospital at the Zhejiang University School of Medicine in Zhejiang, China, from February 2014 to September 2015, were included in our study. There were 18 males, 12 females, and the median age was 53.5 years old. All of the patients were treated with doxorubicin-loaded HepaSphere microspheres. Each of the HCC patients was diagnosed clinically and pathologically [27], and all of the patients with liver metastasis had a biopsy-confirmed pathological diagnosis. Based on the tumor vascularity and pathology, 16 of the patients were classified as having hypervascular tumors (all had HCC). Fourteen of the patients had hypovascular tumors: three had metastatic colorectal cancer (mCRC), two had metastatic gallbladder tumors, three had metastatic neuroendocrine tumors, two had metastatic cholangiocarcinoma, two had metastatic pancreatic carcinoma, one had a metastatic lung tumor, and one had metastatic gastric cancer.

Letters of consent were obtained from all of the patients, and the experimental protocols were approved by the local ethics committee (The Medical Ethics Committee of the First Affiliated Hospital, Zhejiang University School of Medicine, Zhejiang, China). The procedure was carried out in accordance with the approved guidelines, and informed consent was obtained from all of the subjects. All of the data was anonymized and de-identified prior to the analysis.

### Patient eligibility

### Inclusion criteria

A patient was included in this study if they: (1) had an unresectable HCC or liver metastasis, were not a candidate for locoregional tumor ablation, were refractory or intolerant of systemic chemotherapy, or had recurrent HCC; (2) were Child-Pugh status A or B, or Eastern Cooperative Oncology Group (ECOG) 0 to 2; and (3) did not have a tumor thrombosis in the main portal vein.

### Exclusion criteria

A patient was excluded if they: (1) were Child-Pugh status C, (2) had uncorrectable impaired clotting, (3) had a tumor thrombosis in the main portal vein or portal hypertension, (4) had an infectious disease (e.g. liver abscess), (5) had a life expectancy of less than 3 months, or (6) had an aneurysm in the hepatic artery or portal vein.

### Doxorubicin loading and TACE procedure

HepaSphere microspheres are loadable microspheres with a dry caliber of 50-100 μm, which expands to 200-400 μm after doxorubicin loading. The drug loading method was conducted as suggested by the manufacturer. The doxorubicin solution was prepared by adding 4 ml of normal saline into each 10 mg vial of doxorubicin powder, creating a concentration of 2.5 mg/ml. Five 20 ml vials of doxorubicin were prepared for each patient. Next, 10 ml of the doxorubicin solution was added into a 50-100 μm HepaSphere vial, mixed gently 5-10 times, and allowed to sit for 10 minutes. After 10 minutes, the remaining doxorubicin solution was loaded into the HepaSphere vial and allowed to stand for 60 minutes. An hour later, all of the supernatant was extracted, and 20 ml of nonionic contrast medium was added into a syringe containing the doxorubicin-loaded HepaSphere microspheres, and mixed gently until homogeneity was reached.

### Procedure

The interventional procedure was done by the experienced interventional radiologists at our hospital. Hepatic angiography was performed to identify the feeding artery and collaterals. In the case of a hypovascular tumor, angiography at the superior mesenteric artery, inferior mesenteric artery, and left gastric artery had to be conducted.

The TACE procedures followed the classical steps, and were performed superselectively in the tumor-feeding arteries. After the identification of the feeding arteries, a microcatheter was advanced as distally as possible. Then, the HepaSphere microspheres were injected slowly, at a rate of 1 ml/min, until stasis was observed. The angiography was repeated to ensure the devascularization of the feeding arteries had occurred. In those cases in which a tumor stain was observed, the HepaSphere microspheres were injected until the tumor stain disappeared. Normally, one vial of HepaSphere was required, with a maximum dose of two vials. Embosphere microspheres (Merit Medical Systems, Inc., South Jordan, UT, USA) were injected into the feeding arteries when the tumor stain was still observed after two HepaSphere vials were used.

### Postembolization patient management

The routine postembolization medication included the following: 100 mg of tramadol was only used in case of pain, 40 mg of esomeprazole was given intravenously every 12 h, 5 mg of ondansetron was given intravenously every 12 h, and 1500 mg cefuroxime sodium was given intravenously every 12 h for 3 days after embolization. Intravenous drops of 80 mg of a glycyrrhizin compound were given daily, with 1000 mg of ademetionine to protect the liver function. Finally, 200 mg of celecoxib was used if the temperature was higher than 38.5°C.

### Follow-up

CT/MRI images were assessed one month after the procedure using the modified Response Evaluation Criteria in Solid Tumors (mRECIST) [7]. Laboratory analyses, including 3-day liver function tests for each patient, were also performed. During the procedure and follow-up, any adverse events were collected according to the National Cancer Institute Common Toxicity Criteria (NCI-CTC) ver. 4.0.

### Data analysis

The data processing and analyses were performed using SPSS 19.0 (Chicago, IL, USA). Chi-squared and t-tests were used when appropriate, and statistical significance was defined as a *P* value <0.05.
